# Human bocavirus 1 coinfection is associated with decreased cytokine expression in the rhinovirus‐induced first wheezing episode in children

**DOI:** 10.1002/clt2.12311

**Published:** 2023-11-13

**Authors:** Pekka Hurme, Reetta Sahla, Beate Rückert, Tero Vahlberg, Riitta Turunen, Tytti Vuorinen, Mübeccel Akdis, Maria Söderlund‐Venermo, Cezmi Akdis, Tuomas Jartti

**Affiliations:** ^1^ Department of Pediatrics and Adolescent Medicine Turku University Hospital University of Turku Turku Finland; ^2^ Swiss Institute of Allergy and Asthma Research (SIAF) University of Zürich Christine Kühne‐Center for Allergy Research and Education (CK‐CARE) Davos Switzerland; ^3^ Department of Biostatistics University of Turku Turku Finland; ^4^ New Children's Hospital Helsinki University Hospital University of Helsinki Helsinki Finland; ^5^ Institute of Biomedicine University of Turku Turku Finland; ^6^ Department of Clinical Microbiology Turku University Hospital Turku Finland; ^7^ Department of Virology University of Helsinki Helsinki Finland; ^8^ Research Unit of Clinical Medicine Medical Research Center University of Oulu Oulu Finland; ^9^ Department of Pediatrics and Adolescent Medicine Oulu University Hospital Oulu Finland

**Keywords:** bocavirus, bronchiolitis, cytokine, rhinovirus, virus, wheeze, wheezing

## Abstract

**Background:**

Rhinovirus (RV)‐induced first wheezing episodes in children are associated with a markedly increased risk of asthma. Previous studies have suggested that human bocavirus 1 (HBoV1) may modify RV‐induced immune responses in young children. We investigated cytokine profiles of sole RV‐ and dual RV‐HBoV1‐induced first wheezing episodes, and their association with severity and prognosis.

**Methods:**

Fifty‐two children infected with only RV and nine children infected with dual RV‐HBoV1, aged 3–23 months, with severe first wheezing episodes were recruited. At acute illness and 2 weeks later, peripheral blood mononuclear cells were isolated, and stimulated with anti‐CD3/anti‐CD28 in vitro. Multiplex ELISA was used to quantitatively identify 56 different cytokines at both study points. Patients were prospectively followed for 4 years.

**Results:**

The mean age of the children was 14.3 months, and 30% were sensitized. During the acute illness, the adjusted analyses revealed a decrease in the expression of IL‐1b, MIP‐1b, Regulated upon activation, normal T cell expressed and presumably secreted (CCL5), TNF‐a, TARC, and ENA‐78 in the RV‐HBoV1 group compared with the RV group. In the convalescence phase, the RV‐HBoV1 group was characterized by decreased expression of Fractalkine, MCP‐3, and IL‐8 compared to the RV group. Furthermore, the hospitalization time was associated with the virus group and cytokine response (interaction *p* < 0.05), signifying that increased levels of epidermal growth factor and MIP‐1b were related with a shorter duration of hospitalization in the RV‐HBoV1 coinfection group but not in the RV group.

**Conclusions:**

Different cytokine response profiles were detected between the RV and the RV‐HBoV1 groups. Our results show the idea that RV‐induced immune responses may be suppressed by HBoV1.

## 1 INTRODUCTION

Up to 30% of all children suffer from bronchiolitis before the age of two, and it is the leading cause necessitating hospitalization in early childhood.^1^ While the respiratory syncytial virus (RSV) is the most common etiology among children under 12 months of age, rhinovirus (RV) infections dominate thereafter. Human bocavirus (HBoV1) is the next most common virus related to bronchiolitis during the early years of life.^1^, ^2^ In previous studies, the RV etiology of bronchiolitis is linked with a much greater risk of subsequent recurrent wheezing as well as asthma than all other etiologies.^1^, ^2^, ^3^, ^4^, ^5^

Acute RV infection, which targets and replicates in the epithelium of the upper airways, leads to a rapid surge of type I and III interferons, and in the activation of innate immunity.^1^ Several cytokines and chemokines are released as a result, thereby leading to epithelial cell death, necrosis, epithelial sloughing, and excessive mucus production. As opposed to other viral infections, atopic susceptibility, and variations in the cadherin‐related family member 3 gene or 17q21 locus in RV infections, increase the likelihood of a more severe illness and a poorer long‐term prognosis.^5^, ^6^, ^7^ Additionally, data from studies using human and murine models have suggested that RV infections of the airway epithelium induce type 2 innate cytokines, such as IL‐25 and IL‐33, which consequently commence or enhance type 2 immunity in the lungs through activation of the IL‐5‐ and IL‐13‐producing innate lymphoid cells (ILC) 2 and T‐helper 2 (Th2) cells.^8^, ^9^, ^10^

Whereas RV is classified as a nonenveloped single‐stranded RNA virus belonging to the *Picornaviridae* family, HBoV1 is part of the *Parvoviridae* family and is classified as a non‐enveloped single‐stranded DNA virus.^11^ HBoV1 is a common agent found in infections of the respiratory‐tract, for example, bronchiolitis, recurrent wheezing, and asthma in young children.^12^ Data regarding the bocavirus‐associated T‐cell mediated immune responses are scarce.^13^, ^14^, ^15^ However, a few studies have suggested that HBoV1, in coinfection with RV, may have immunomodulatory effects.^13^, ^16^, ^17^ Hence, we aimed to investigate the cytokine profiles of the severe first wheezing episode caused by RV versus RV‐HBoV1 as well as their association with a short‐term prognosis. Our hypothesis is that a difference in cytokine expression from peripheral blood mononuclear cells (PBMCs) of children with virus‐induced acute wheezing due to RV compared with RV‐HBoV1 may be potentially associated with its severity and that HBoV1 coinfection may exhibit immune‐suppressive activity.

## 2 MATERIALS AND METHODS

### 2.1 Subjects

**FIGURE 1:**
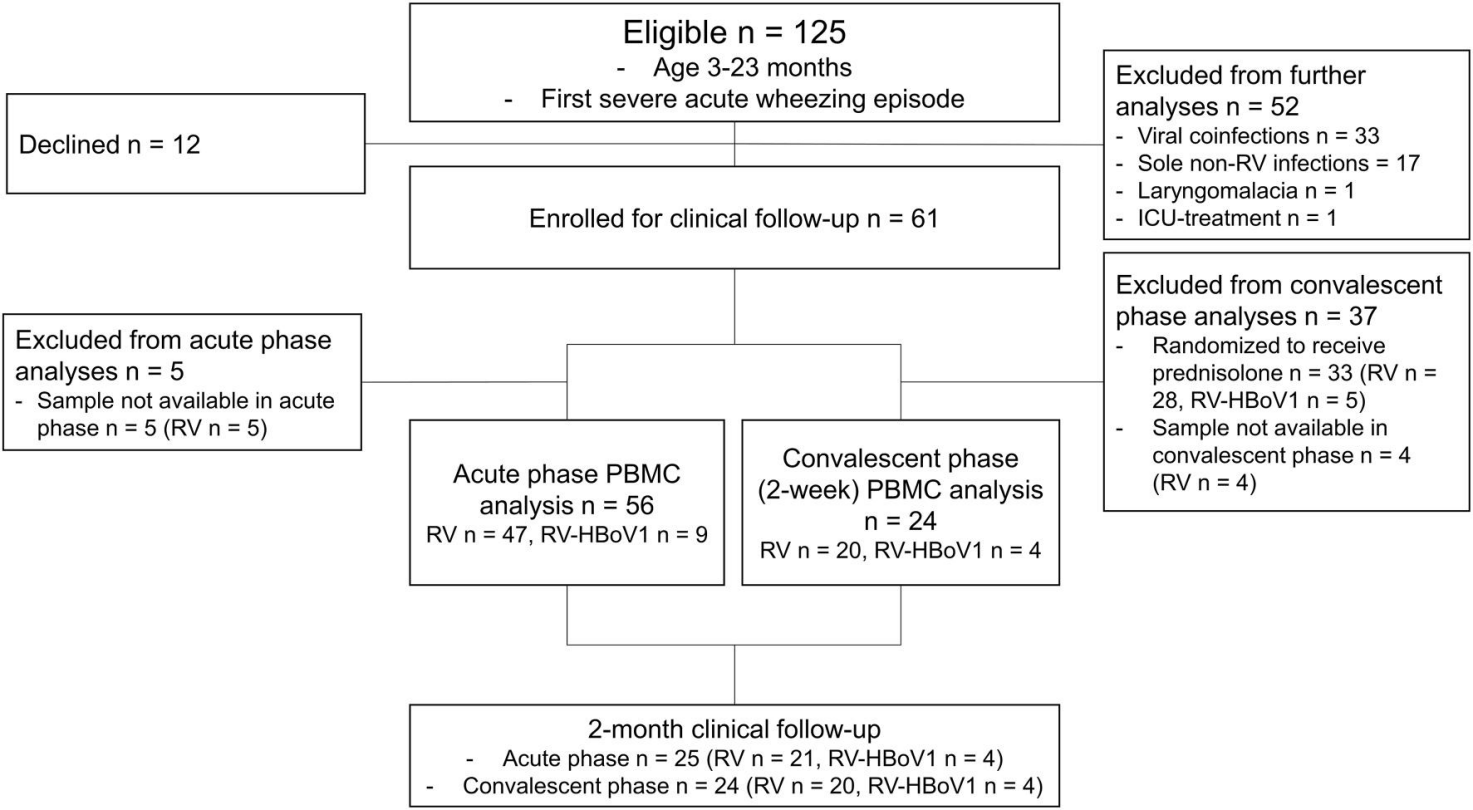
Study flow chart. Patients with cytology data were included. HBoV1, human bocavirus‐1; ICU, intensive care unit; PBMC, peripheral blood mononuclear cell; RV, rhinovirus.

The study population was derived from our previous trial, Vinku2 study, which compared the long‐term efficacy of oral prednisolone (2 mg/kg/d for 3 days) to placebo in RV‐positive first‐time wheezing children (updated version for 7‐year follow‐up, trial no: NCT00731575, original version EudraCT 2006‐007100‐42).^18^ Patients were recruited prospectively in 2007–2010 at the Department of Pediatrics, Turku University Hospital (Turku, Finland). The inclusion criteria included of patient's aged 3–23 months, delivery at ≥36 weeks of gestation, the first episode of wheezing (parental report and verified from medical charts), sole RV infection or RV‐HBoV1 coinfection as evidenced by detection of viral RNA/DNA in a nasopharyngeal aspirate (NPA) sample by polymerase chain reaction (PCR) as well as serology for HBoV1 without prior steroid treatment. A parent or guardian provided written informed consent beforehand. The exclusion criteria were chronic non‐atopic disease, previous corticosteroid treatment, or the need for intensive care (Figure 1, study flow chart). The Ethics Committee of Turku University Hospital approved the study protocol.

### 2.2 Study protocol

The admission to the hospital was determined by an on‐duty physician independent of the study. The recruitment to the study was conducted by the study physician. During the enrollment, the child's guardian was interviewed using a standard survey on risk factors for asthma (host and environmental) and the child was clinically assessed by the study physician. A NPA sample was taken for viral diagnostics via a standardized procedure,^19^ and a blood sample was obtained. After a positive RV‐PCR test, the children were randomized to receive either oral prednisolone or a placebo (prednisolone recipients were excluded from the later analyses). Two weeks after the enrollment, a second blood sample was obtained. The patients reported their symptoms for the first 2 months using daily symptom diaries. Furthermore, episodes of breathing difficulties and medication were documented in a diary, and the children were interviewed during follow‐up visits at 2 weeks, 2 months, 12 months, and 4 years by the study physicians. Furthermore, the children's guardians were advised to revisit the study physician each time the child showed signs and symptoms of difficulty breathing.

### 2.3 Outcomes

1. To compare the cytokine profiles of children suffering from severe first‐time wheezing who were infected with RV‐ and HBoV1 to those with RV only. Samples were taken at acute phase and 2 weeks after the infection.
2. To determine whether the cytokine response is associated with the severity of illness (i.e., duration of hospitalization).
3. To assess whether cytokine expression is associated with occurrence of relapses (at 2‐ and 12‐month follow‐up) and asthma at 4 years.

### 2.4 Definitions

Wheezing was defined as difficulty of breathing accompanied by bilateral high‐pitched whist‐like sounds during expiration. The occurrence of wheezing event combined by RV‐RNA or RV RNA‐HBoV1‐DNA detection by PCR and HBoV1 serology were called RV‐ or RV‐HBoV1‐induced wheezing episodes, respectively. Atopy refers to positive allergen‐specific immunoglobulin E (IgE) antibody (cut‐off level ≥0.35 kU/L) to one or more of the common allergens (codfish, cow's milk, egg, peanut, soybean, wheat, cat, dog, horse, birch, mugwort, timothy, *Cladosporium herbarum*, and *Dermatophagoides pteronyssinus*) (Phadiatop Combi®, Phadia, Uppsala, Sweden). Positive IgE antibodies to one or more of the latter eight allergens were referred to as aeroallergen sensitization. Additionally, positive IgE antibodies against dog, cat, or *Dermatophagoides pteronyssinus* were defined as perennial aeroallergen sensitization. Eczema was diagnosed by typical clinical signs of pruritus, morphology, and longevity of the illness. If the child had atopy and eczema, the latter was defined as atopic eczema (defined above). Further, type 1 immunity is referred as the action of T‐helper 1 cells, type 1 innate lymphoid cells, neutrophils, and classically activated macrophages. Type 2 immunity is referred to as the action of Th2 cells, type 2 innate lymphoid cells (ILC2), eosinophils, mast cells, basophils, and IL‐4‐ and IL‐13‐activated macrophages.

### 2.5 Laboratory data

A NPA was obtained using a nasal swab (nylon flocked dry swab, 520CS01, Copan, Brescia, Italy) and deposited at −70°C. Viral PCR analyses were performed as described earlier.^12^, ^19^, ^20^, ^21^, ^22^ Briefly, nucleic acids were extracted from samples and viral RNA was detected using in‐house reverse transcription (RT)‐PCR for enteroviruses, RSV (A and B), and RV (A, B, and C), and at the Virus Diagnostic Laboratory, Department of Virology, University of Turku.^23^, ^24^ Additionally, we used a multiplex PCR test (Seeplex RV12 ACE Detection, Seegene, Seoul, Korea) for the detection of adenovirus, coronavirus (229E, NL63, OC43, and HKU1), human metapneumovirus, influenza virus (A and B), parainfluenza virus (types 1–3), RSV (A and B), and RV (A and B). The HBoV1 DNA can persist in the airways for weeks and months after acute infections.^13^ Therefore, HBoV1 infections were analyzed using both PCR and serology (IgM and IgG in paired sera) as previously described.^22^, ^25^ To determine the genotype specificity of the IgG for HBoV1, the sera samples were blocked with HBoV2 and HBoV3 antigens.^25^ The allergen‐specific serum IgE levels as well as the absolute eosinophil (B‐Eos) count were measured according to the standard diagnostics of the Central Laboratory of Turku University Hospital. The levels of serum 25‐hydroxyvitamin D (25(OH)D) were determined by means of liquid chromatography‐tandem mass spectrometry at Massachusetts General Hospital (Boston, MA, USA).

Further, peripheral whole blood was collected and PBMCs were isolated during the acute illness and convalescence phase by Ficoll‐Paque™ PLUS (GE Healthcare, Amersham, United Kingdom) density gradient centrifugation according to the manufacturer's instructions. Peripheral blood mononuclear cells were processed and stored as described earlier.^26^ In short, samples were stimulated for 24 h with 0.25 μL of anti‐CD3 (1 μg/mL) and anti‐CD28 (1 μg/mL) (BD Biosciences, Franklin Lakes, NJ, USA). The supernatants were stored at −80°C. After thawing, the samples were analyzed using the multiplex ELISA (Millipore HCYTOMAG‐60K‐36 and HCYP2MAG‐62K‐20 assays) with the Bio‐Plex 200 System using the Bio‐Plex Manager 6.0 Software (Bio‐Rad, Cressier, Switzerland) to determine the levels of 56 different cytokines.

However, in certain samples, the multiplex fluorescence was below the limit or exceeded the limit of quantification of the assay (Supplementary Table S1). Therefore, a few cytokine measurements did not reach the quantitative limit of detection. To ensure that the conclusions were drawn from a majority of the samples, we decided to only include cytokines that were detected (within the limit of quantification) in more than 50% of the samples for analyses (29/56, 52%). If a sample had a value below the limit of detection, they would be set to half the value of the lower threshold of the assay.^26^, ^27^, ^28^ On the other hand, samples exceeding the limit of detection were assigned to the upper threshold of the assay.^26^, ^29^ The minimum and maximum quantifiable values of the multiplex assays are presented in Supplementary Tables S2 and S3.

### 2.6 Statistics

We tested the data distribution using the Kolmogorov‐Smirnov test. Due to the skewness of the data, we adjusted the cytokine levels using log10 or x^2^ transformation when appropriate. For other statistics, we used the two‐sample *t*‐test, Mann‐Whitney *U* test, χ^2^ test, or Fisher's exact test. Additionally, we used a multivariable linear model to analyze the differences in cytokine expression between study groups after adjustment for baseline characteristics. For the final adjusted model, the backward stepwise method was used separately for each cytokine (inclusion criteria *p* < 0.05). To analyze the effects of the viral group (RV vs. RV‐HBoV1) and cytokine expression on the duration of hospitalization, negative binominal regression was used (JMP version 13.1.0, SAS Institute). A more detailed version of the statistics is provided in the Supplementary material.

## 3 RESULTS

### 3.1 Study population

Initially, we enrolled 125 children in the study, of which 12 declined to continue, and 113 proceeded with the clinical follow‐up. Before further analyses, we excluded all patients with infections other than sole RV or dual RV‐HBoV1‐infection. Thus, 61 children were eligible in the acute phase. Next, 5 children were excluded due to the absence of cytology samples, and eventually, we included cytology data from 56 children in the analysis. During the convalescence phase, 33 children had been randomized for prednisolone, and therefore, they were excluded from further analyses. Ultimately, we analyzed cytokines and chemokines of 56 samples in the acute phase, and 24 samples at 2 weeks. Clinical data were available for 25 acute‐ and 24 convalescence‐phase children at 2 months (Figure 1).

### 3.2 Patient characteristics

The mean age of the patients was 14.3 months (SD 5.6), and 73% of the study participants were males. Moreover, 75% were hospitalized, 30% were atopic, and 24% had atopic eczema. RV‐infected children were younger and had fewer signs and symptoms (wheezing, cough, and fever) (all *p* < 0.05, Table 1). Therefore, we adjusted the cytokine analyses to the variables above using the backward stepwise method.

**TABLE 1:** Patient characteristics at study entry.

Characteristic	RV (*n* = 47)	RV‐HBoV1 (*n* = 9)	*p*‐value
Age, months	13.5 (8.8–16.8)	15.4 (14.9–21.7)	**0.03**
Male sex, no.	35 (74%)	6 (67%)	0.69
Weight, kg	10.5 (2.1)	11.3 (1.5)	0.27
Preceding wheezing, days	1 (1–1)	2 (1.5–2.5)	**0.001**
Preceding cough, days	2 (2–3)	5 (3–13.5)	**0.003**
Preceding rhinitis, days	3 (2–5)	5 (0–10)	0.46
Preceding temperature over 37.5 C˚	1 (0–2)	2 (1–2.5)	**0.03**
Clinical score, points	5 (4–8)	5 (4–8)	0.99
Oxygen saturation, %	97 (95–98)	97 (95–99)	0.35
Temperature, C˚	37.4 (0.57)	37.7 (0.82)	0.31
CRP, mg/L	13 (6–21)	9 (5–22)	0.55
B‐Eos (1 × 10^9^/L)	0.52 (0.35–0.73)	0.46 (0.31–0.86)	0.79
B‐Eos >0.4 × 10^9^/L	29 (64%)	5 (56%)	0.71
Total IgE	22 (9–48)	23 (11–64)	0.78
Eczema, no.	10 (22%)	3 (33%)	0.43
Atopic eczema, no.	10 (22%)	3 (33%)	0.43
Sensitization, no.	15 (33%)	3 (33%)	0.99
Food, no.	14 (30%)	3 (33%)	0.79
Aero, no.	10 (22%)	1 (11%)	0.67
Perennial, no.	9 (20%)	1 (11%)	0.96
Parental asthma, no.	9 (19%)	2 (22%)	0.83
Parental allergy, no.	29 (62%)	8 (89%)	0.11
Parental smoking, no.	22 (47%)	4 (44%)	0.90
S‐25‐OHD, nmol/L	84 (72–99)	80 (61–95)	0.48

*Note*: Values are presented as mean (SD), median (interquartile range), or number (%). Data were analyzed using the two‐sample *t*‐test, Mann‐Whitney *U*‐test, χ^2^ test or Fisher exact test. Bold text; Statistical significance *p* < 0.05.

Abbreviations: B‐Eos, blood eosinophil count; HBoV1, Human Bocavirus‐1; RV, rhinovirus; S‐25‐OHD, serum 25‐hydroxyvitamin D.

### 3.3 Differences in cytokine expression at study entry

**FIGURE 2:**
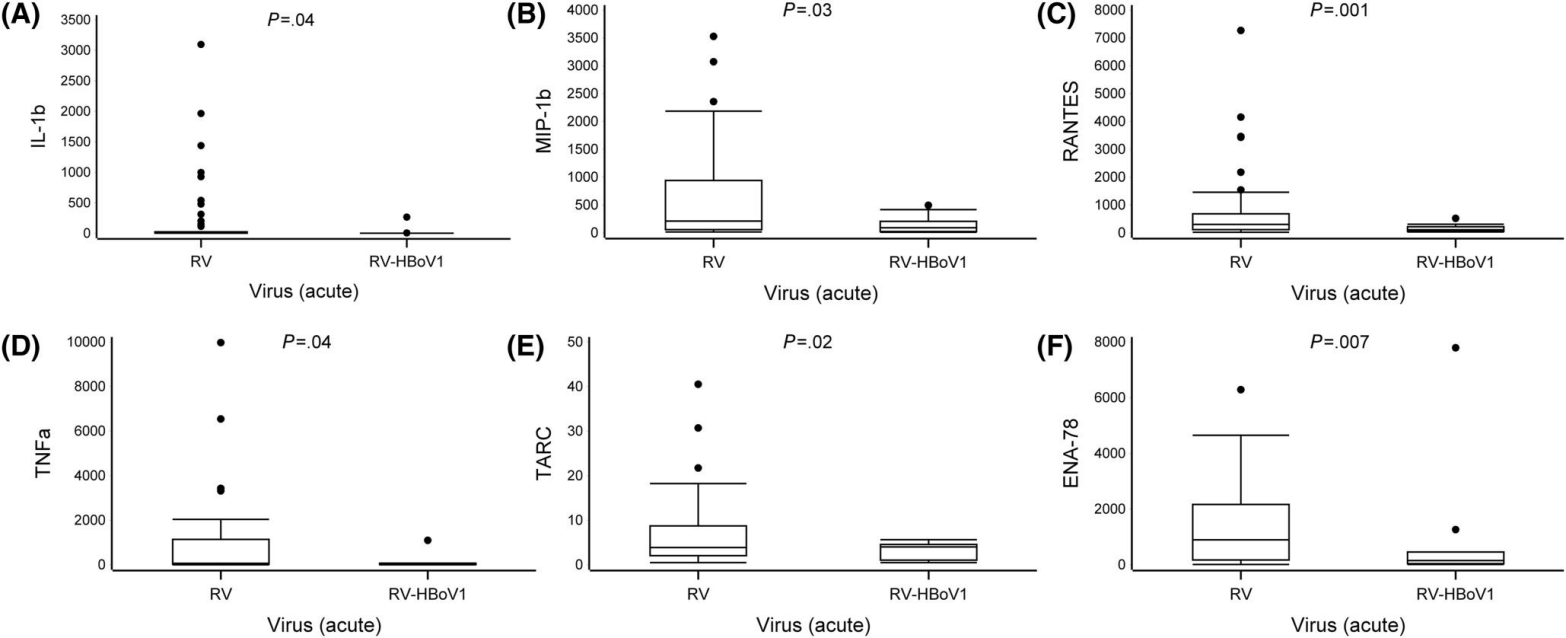
Differences in cytokine expression levels at the study entry. Data are presented as medians (the lower (Q1) and upper (Q3) quartiles, and data falling outside the Q1–Q3 range are plotted as outliers). Cytokine concentrations are presented as pg/mL. In the difference in cytokine expression, multiple significant differences were observed between the two virus groups (rhinovirus (RV) vs. RV‐HBoV1, all *p* < *0.05*) (A–F).

After stimulation with anti‐CD3 and anti‐CD28, we observed significant differences in cytokine responses between the patients infected with RV and those infected with RV‐HBoV1. In the acute phase, the RV‐HBoV1 group had lower expression of IL‐1b (median 1.6 vs. 3.5 pg/mL), MIP‐1b (92 vs. 210), Regulated upon activation, normal T cell expressed and presumably secreted, CCL5 (RANTES) (110 vs. 300), TNFa (33 vs. 65), TARC (1.9 vs. 4.4) and ENA‐78 (150 vs. 900) as compared to the RV group (all *p* < 0.05, Table 2, Figure 2, Supplementary Table S4). We describe the biological functions of these cytokines in Supplementary Table S5.

**TABLE 2:** Differences in cytokine expression levels at study entry.

Cytokine	Timing	RV	RV‐HBoV1	*p*‐value, univariate	*p*‐value, multivariate	Adjustments
		*n*(acute) = 47	*n*(acute) = 9			
		n(convalescent) = 20	n(convalescent) = 4			
Fractalkine	Acute	14 (8.3–28)	13 (7.8–21)	0.64	0.59	‐
	Convalescent	15 (9.3–21)	9.9 (3.6–15)	0.11	**0.04**	‐
MCP‐3	Acute	300 (120–390)	99 (44–660)	0.56	0.59	‐
	Convalescent	390 (36–390)	10 (6.2–64)	**0.03**	**0.01**	2
IL‐1b	Acute	3.5 (1.6–25)	1.6 (1.6–4.7)	0.13	**0.04**	1
	Convalescent	25 (1.6–270)	1.6 (1.6–15)	0.09	0.12	‐
IL‐6	Acute	32 (6.8–380)	7.0 (1.6–27)	0.09	0.13	‐
	Convalescent	66 (4.2–2000)	1.6 (1.6–36)	**0**.**049**	0.06	‐
IL‐8	Acute	1400 (650–2500)	1000 (430–1800)	0.51	0.63	‐
	Convalescent	840 (600–1600)	240 (120–660)	**0.02**	**0.02**	‐
MIP‐1b	Acute	210 (58–940)	92 (19–310)	**0.03**	**0.03**	‐
	Convalescent	430 (48–1300)	30 (17–280)	0.06	0.07	‐
RANTES	Acute	300 (120–680)	110 (47–260)	**0.03**	**0.001**	1,3
	Convalescent	250 (69–800)	53 (22–530)	0.16	0.15	2
TNFa	Acute	65 (25–1100)	33 (14–69)	0.14	**0.04**	1
	Convalescent	420 (11–1300)	12 (4.9–620)	0.24	0.23	‐
TARC	Acute	4.4^a^ (3.3–5.9)	1.9^a^ (1.0–3.6)	0.19	**0.02**	1
	Convalescent	4.1 (2.4–7.8)	2.0 (1.9–5.7)	0.25	0.51	‐
ENA‐78	Acute	900 (170–2200)	150 (25–860)	0.054	**0.007**	2
	Convalescent	190 (65–1800)	78 (23–150)	0.18	0.62	2,3

*Note*: Acute sample, samples drawn at the study entry; Convalescence sample, samples drawn at 2‐week follow‐up. Values are presented as medians (interquartile range). Data were analyzed using the Mann‐Whitney *U*‐test, and the multivariable linear model. Log_10_‐transformed cytokine expression levels were used in the analyses. The adjustments for immunologic analyses included baseline characteristics that significantly differed between the groups (Age = 1, and duration of previous symptoms (cough = 2, wheezing = 3, fever = 4) at entry). A backward stepwise method was used for the final adjustment model separately for each cytokine. Only statistically significant baseline characteristics variables (*p* < 0.05) were included in the final model. All data are presented in Supplementary Table S4. Bold text; Statistical significance *p* < 0.05.

^a^ Age‐adjusted geometric means (95% confidence intervals).

### 3.4 Differences in cytokine expression in the convalescence phase

**FIGURE 3:**
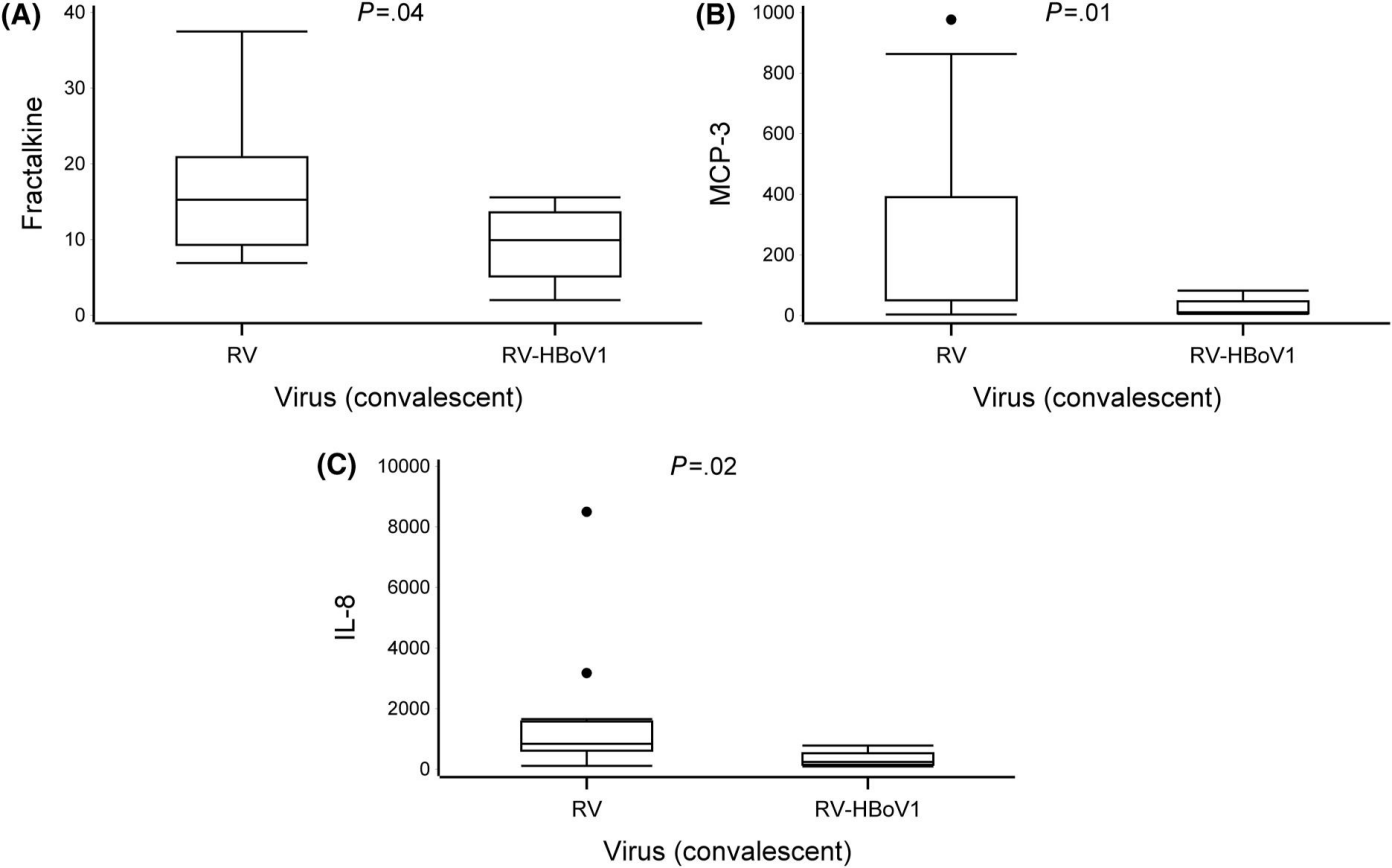
Differences in cytokine expression levels at convalescence phase. Data are presented as medians (the lower (Q1) and upper (Q3) quartiles, and data falling outside the Q1–Q3 range are plotted as outliers). Cytokine concentrations are presented as pg/mL. In the difference in cytokine expression, multiple significant differences were observed between the two virus groups (rhinovirus (RV) vs. RV‐HBoV1, all *p* < *0.05*) (A–C).

In the convalescence phase, we observed lower expression of fractalkine (median 9.9 vs. 15 pg/mL), MCP‐3 (10 vs. 300), and IL‐8 (240 vs. 840) in the RV‐HBoV1 group in comparison to the RV group (all *p* < 0.05, Table 2, Figure 3, Supplementary Table S4). The expression of IL‐6 and MIP‐1b was marked but not statistically significant (all *p* < 0.07, Table 2, Supplementary Table S4). No statistically significant difference was found in the change in cytokine expression between the acute and convalescence phases when comparing the study groups (all *p* > 0.05, Supplementary Table S4).

### 3.5 The association between cytokine expression and the severity of acute illness

The duration of hospitalization correlated with the virus group and cytokine response. We observed two statistically significant interactions between the virus groups and the cytokine response (all *p* < 0.04, Table 3, Supplementary Table S6), thereby signifying that the effect of cytokine response on hospitalization time differed when comparing the two study groups. Further, an increased expression of epidermal growth factor (EGF) and MIP‐1b was associated with a shorter duration of hospitalization in the RV‐HBoV1 group (all *p* < 0.03) but not in the RV group (all *p* > 0.11, Table 3, Supplementary Table S6).

**TABLE 3:** Association between cytokine expression and severity of acute illness (duration of hospitalization).

Outcome duration of hospitalization	Group effect RV versus RV‐HBoV1		Cytokine effect expression of cytokine		Group × cytokine interaction effect
	Estimate (95% CI)	*p*‐value	Estimate (95% CI)	*p*‐value	*p*‐value
EGF	e	e	1.090^a^ (0.629, 1.883)	0.76^c^	**0.04**
			0.192^b^ (0.064, 0.579)	**0.003** ^d^	
MIP‐1b	e	e	1.200^a^ (0.958, 1.503)	0.11^c^	**0.03**
			0.588^b^ (0.370, 0.933)	**0.024** ^d^	

*Note*: Data were using the negative binominal regression with log‐transformed cytokine level. Log_10_‐transformed cytokine expression levels were used in the analyses. All data are presented in Supplementary Table S6. Bold text; Statistical significance *p* < 0.05.

Abbreviations: CI, confidence interval; EGF, epidermal growth factor.

^a^ Relative risk: RV group‐negative binomial regression.

^b^ Relative risk: RV‐HBoV1 group‐negative binomial regression.

^c^ Group effect in RV arm.

^d^ Group effect in RV‐HBoV1 arm.

^e^ Due to the significant interactions, the cytokine effect was not estimated using all data. The effect of cytokine is presented separately in the RV and the RV‐HBoV1 groups.

### 3.6 The association between cytokine expression in both recurrencies and asthma

The occurrence of relapses within 2 and 12 months after the infection did not differ between the RV and RV‐HBoV1 groups (52% [11/21] vs. 0% [0/4], *p* = 0.10, and 81% [17/21] vs. 25% [1/4], *p* = 0.053, respectively). Unfortunately, due to the small number of children in the RV‐HBoV1 group, we could not assess the association between cytokine expression and the incidence of recurrent wheezing within 2 months, 12 months, and asthma 4 years after the infection (data not shown).

## 4 DISCUSSION

In this study, we investigated the dynamics of multiple cytokines and chemokines from stimulated PBMCs in young first‐time wheezing children during and after sole RV versus dual RV‐HBoV1 infection. We demonstrated 1) lower cytokine responses in RV‐HBoV1 infection compared to sole RV infection during the acute illness, and the convalescent phase; and 2) that increased levels of EGF and MIP‐1b were associated with less severe disease in the RV‐HBoV1 group.

Although, the RV and the RV‐HBoV1 groups showed resemblances in the general cytokine response during the acute illness, the RV‐HBoV1 group showed more of a decreasing trend throughout all cell‐mediated immunity subtypes (types 1 and 2) as well as a decreasing pro‐inflammatory cytokine profile. Distinctions of the different subtypes of cell‐mediated immunity have been described in detail before.^30^ During the acute illness, children with RV‐HBoV1 coinfection were characterized by lower expressions of IL‐1b, MIP‐1b, RANTES, TNFa, TARC, and ENA‐78. IL‐1b and TNFa are pro‐inflammatory cytokines, known for their crucial role in host defense in response to infections and injuries, in the induction of proinflammatory proteins, as well as in the promotion of differentiation of Th17 cells.^31^, ^32^ MIP‐1b and RANTES are generally classified as type 2 associated chemokines, known for recruitment of eosinophils, for example, in the airways.^33^, ^34^ However, in recent studies, the latter have been suggested to have more pleiotropic capabilities.^35^ Moreover, in RV‐affected subjects, RANTES has been associated with bronchial smooth muscle cell chemotaxis, thereby contributing to possible airway remodeling.^36^ TARC distinguishes itself by selectively binding to C‐C chemokine receptor type 4, thereby resulting in induction the of type 2 immune responses via, for example, ILC2, Th2 cells, and airway eosinophils.^37^ ENA‐78 is primarily a neutrophil chemoattractant, and counterintuitively, the expression of ENA‐78 was higher in children infected with RV.^38^

In the convalescence phase, the former differences observed in the acute phase were dissipated. However, in the convalescence phase (2 weeks later) the RV‐HBoV1 group was demonstrated by decreased expression of fractalkine, MCP‐3, and IL‐8 when compared with the RV group. Fractalkine is known for inducing chemotaxis and it has antiviral properties,^39^ whereas MCP‐3 has been suggested as a profibrotic chemokine,^40^ that is associated with activation and migration of monocytes and macrophages in the airways.^41^ IL‐8, known for the robust chemotaxis of neutrophils in the airways, activates the inflammatory cells via the recruitment of innate and adaptive immunity cells. Moreover, IL‐8 is also suggested to participate in airway remodeling in asthmatic patients.^42^, ^43^, ^44^ Overall, when analyzing the change in cytokine expression between the study points, no difference was found. We describe the biological functions of these cytokines in Supplementary Table S5.

Although, the cytokine response was not related to the severity of disease in the RV group, higher expression of EGF, and MIP‐1b in the RV‐HBoV1 group were associated with a shorter duration of hospitalization. EGF plays a significant role in the regulation and in the development of the integrity of the intestinal barrier and homeostasis of mucosa, and interestingly, lower systemic EGF responses have previously been suggested to be associated with a higher prevalence of atopic IgE responses.^45^ Therefore, children infected with RV‐HBoV1 may exhibit a more competent resolution of the disease. MIP‐1 beta is a type 1 associated cytokine; in adult asthma patients, a higher MIP‐1 beta level has been associated with a poorer response to anti‐IL‐5 therapy.^46^ Moreover, a previous study in adult asthmatic patients showed decreased intracellular responses of MIP‐1 beta from CD4+ and CD8+ T‐cells after stimulation in vitro compared to nonasthmatic controls.^47^ Thus, reflecting immunity polarization away from type 1 immunity. Our findings are in line with these results.

Wheezing illness induced by RV infection, at least partially, has numerous asthma‐like features, both clinically and pathophysiologically, as described earlier.^1^ Though, in general, the expression of the cytokines in both study groups resembled each other, HBoV1 coinfection appeared to suppress the overall cytokine profile (type 1, and type 2, as well as a proinflammatory profile) compared to sole RV infection. Interestingly, during the convalescence phase these former differences seemed to disappear. Surprisingly, in the convalescence phase (2 weeks later), new additional differences in cytokine expression could be observed between the study groups. We describe the biological functions of these cytokines in Supplementary Table S5.

The co‐detection of HBoV1 with other respiratory viruses is common due to prolonged persistence of HBoV1 DNA; hence, the diagnosis of acute HBoV1 infection should be done via serology, quantitative PCRs, detecting viral mRNA or detection of high copies of DNA in respiratory samples or serum.^12^, ^13^ Previous studies from nasal swabs and tonsil tissue samples have shown decreased T‐cell‐mediated immune responses with regard to HBoV1 infection.^48^ Furthermore, B and T cells and monocytes in tonsillar germinal centers have been shown to harbor HBoV1 DNA with unknown consequences.^49^ Our previous study on serum samples revealed that HBoV1 modulates the RV‐associated immune response in wheezing children.^16^ These findings are in line with our current results.

Although coinfections in respiratory illnesses such as bronchiolitis are common, data concerning the possible virus‐virus interactive effect in RV‐induced bronchiolitis is relatively scarce because most previous studies have included the coinfections in the analyses.^1^ Thus, in this setting, the impact of viral interaction in specific coinfections has remained unclear. Interestingly, one recent study showed interaction between RSV and influenza A virus, resulting in hybrid virus particles indicating interaction between the viral agents.^50^ In this study, we demonstrated that acute wheezing illnesses induced by sole RV and dual RV‐HBoV1 may exhibit different pathogenetic mechanisms.

Although, activation by anti‐CD3 and anti‐CD28 strongly imitates physiological activation of T cells mediated by T‐cell receptor,^51^ additional differences in stimulation protocols may result in an alteration in cytokine responses. Thus, comparing the results of other studies conducted under different stimulation protocols with ours is challenging, and the confirmation of our findings should be performed within similar settings. Surprisingly, a few of the statistically significant differences in cytokine response did not derive from T‐cells. However, though anti‐CD3 and anti‐C28 stimulation leads to direct activation of T cells, it may indirectly activate other classes of lymphocytes, which results in increased expression of cytokines of non‐T cell derivation.^52^ The cytokine panels in our study were comprehensive and able to measure several inflammatory responses, not only T‐cell‐derived events. This, in turn, was partially deliberate, as the current study design and protocol are novel; hence, we could not anticipate which cytokine differences or responses to expect.

Our current study has many strengths, including thoroughly selected study subjects as well as comprehensive viral diagnostics and detailed analyses of cytokine responses. The original hypothesis of our current study was to distinguish the two viral illnesses from each other; thus, we did not have a “control” group. In addition, our study also has a few limitations. First, statistical power analyses were not conducted, and the relatively small number of participants did not allow optimal multivariable model analysis. However, both viral groups comprised thoroughly selected novel subgroups of bronchiolitis. Second, a limited volume of culture medium prevented the ability to perform the dilution series and thus the fluorescence of a few cytokines surpassed the upper limit of quantification and complicated the cytokine analyses as a result. However, the number of these cytokines was relatively small (Supplementary Table S1). Third, the cytokine expression from stimulated PBMCs may be different from the expression in other parts of the body, for example, the airways. Lastly, although early wheezing illness induced by RV has been linked with much greater risk of subsequent recurrent wheezing as well as asthma compared to other viral etiologies,^5^ we were unable to assess the long‐term prognosis for the RV‐HBoV1 group due to relatively small sample size.

In summary, our current study supports the emerging assumption that HBoV1 coinfection may suppress cytokine expression in the RV‐induced first wheezing episode in children, as manifested by alterations in the overall cytokine and chemokine profiles. Our findings also suggest potential new biomarkers for early events of asthma in high‐risk cohorts, predominantly first‐time wheezing children infected with RV or RV‐HBoV1. Our study also suggests that viral proteins are likely to act as potent immunomodulators. However, further trials are warranted.

## AUTHOR CONTRIBUTIONS

**Pekka Hurme**: Conceptualization (equal); Data curation (equal); Formal analysis (lead); Investigation (lead); Software (lead); Validation (equal); Visualization (lead); Writing – original draft (lead); Writing – review & editing (equal). **Reetta Sahla**: Investigation (equal); Validation (equal); Visualization (equal); Writing – original draft (equal); Writing – review & editing (equal). **Beate Rückert**: Data curation (equal); Methodology (equal); Resources (supporting); Software (supporting); Visualization (supporting); Writing – original draft (supporting); Writing – review & editing (supporting). **Tero Vahlberg**: Data curation (supporting); Formal analysis (supporting); Methodology (supporting); Software (equal); Supervision (supporting); Validation (supporting); Visualization (equal); Writing – original draft (supporting); Writing – review & editing (supporting). **Riitta Turunen**: Data curation (supporting); Investigation (equal); Methodology (supporting); Resources (supporting); Writing – original draft (supporting); Writing – review & editing (supporting). **Tytti Vuorinen**: Conceptualization (supporting); Data curation (supporting); Funding acquisition (supporting); Investigation (supporting); Methodology (supporting); Project administration (supporting); Resources (supporting); Supervision (equal); Validation (equal); Writing – original draft (supporting); Writing – review & editing (supporting). **Mübeccel Akdis**: Investigation (supporting); Methodology (supporting); Project administration (supporting); Resources (equal); Supervision (equal); Validation (supporting); Writing – original draft (supporting); Writing – review & editing (supporting). **Maria Söderlund‐Venermo**: Data curation (supporting); Formal analysis (supporting); Investigation (equal); Methodology (supporting); Project administration (supporting); Resources (supporting); Supervision (equal); Validation (supporting); Writing – original draft (supporting); Writing – review & editing (supporting). **Cezmi Akdis**: Investigation (supporting); Methodology (supporting); Project administration (equal); Resources (equal); Supervision (equal); Validation (supporting); Writing – original draft (supporting); Writing – review & editing (supporting). **Tuomas Jartti**: Conceptualization (equal); Data curation (supporting); Formal analysis (supporting); Funding acquisition (lead); Investigation (equal); Methodology (supporting); Project administration (lead); Resources (equal); Software (supporting); Supervision (lead); Validation (lead); Visualization (supporting); Writing – original draft (supporting); Writing – review & editing (equal).

## CONFLICT OF INTEREST STATEMENT

The authors declare no conflicts of interest in connection with this paper.

## Supporting information

Supplementary Material

## Data Availability

Research data are not shared.
