# Supplementary material for: Human bocavirus 1 coinfection is associated with decreased cytokine expression in the rhinovirus‐induced first wheezing episode in children
Source: Clin Transl Allergy. 2023 Nov 13;13(11):e12311. doi: 10.1002/clt2.12311 (PMC10642552; doi:10.1002/clt2.12311)
Supplement: Supplementary file 1 — Supplementary Material [file CLT2-13-e12311-s001.docx]

**Supplementary Table 1.** The quantification of the cytokines

| Cytokine | Acute phase n=97 | | | | | Convalescent phase n=91 | | | | |
| --- | --- | --- | --- | --- | --- | --- | --- | --- | --- | --- |
|  | Within range | OOR < | OOR > | * | *** | Within range | OOR < | OOR > | * | *** |
| EGF | 55 (57%) | 17 |  | 25 |  | 32 (35%) | 21 |  | 38 |  |
| FGF-2 | 39 (40%) | 57 |  | 1 |  | 26 (29%) | 65 |  |  |  |
| Eotaxin | 12 (12%) | 46 |  | 39 |  | 11 (125) | 36 |  | 44 |  |
| TGF-a | 14 (14%) | 63 |  | 20 |  | 12 (13%) | 59 |  | 20 |  |
| G-CSF | 75 (77%) | 20 |  | 1 |  | 64 (70%) | 25 |  | 2 |  |
| GM-CSF | 53 (55%) | 10 |  | 23 | 11 | 45 (49%) | 20 |  | 15 | 10 |
| Fractalkine | 71 (73%) | 23 |  | 1 | 2 | 72 (79%) | 19 |  |  |  |
| IFNa2 | 73 (75%) | 9 |  | 14 | 1 | 47 (52%) | 26 |  | 18 |  |
| IFN-g | 57 (59%) | 12 |  | 28 |  | 52 (57%) | 19 |  | 20 |  |
| IL-10 | 85 (88%) | 1 |  | 11 |  | 75 (82%) | 3 |  | 13 |  |
| MCP-3 | 71 (73%) | 1 | 25 |  |  | 60 (66%) | 1 | 27 | 3 |  |
| IL-12P40 | 31 (32%) | 53 |  | 13 |  | 31 (34%) | 50 |  | 10 |  |
| MDC | 91 (94%) |  | 2 | 4 |  | 87 (96%) | 1 | 1 | 2 |  |
| IL-12P70 | 26 (27%) | 25 |  | 46 |  | 27 (30%) | 27 |  | 37 |  |
| IL-13 | 64 (66%) | 23 |  | 10 |  | 58 (64%) | 24 |  | 9 |  |
| IL-15 | 3 (3%) | 77 |  | 17 |  | 5 (5%) | 73 |  | 13 |  |
| sCD40L | 42 (43%) | 36 |  | 19 |  | 35 (38%) | 40 |  | 16 |  |
| IL-17A | 34 (35%) | 59 |  | 4 |  | 22 (24%) | 61 |  | 8 |  |
| IL-1RA | 95 (98%) | 1 |  | 1 |  | 85 (93%) | 2 |  | 4 |  |
| IL-1a | 36 (37%) | 40 |  | 21 |  | 37 (41%) | 42 |  | 12 |  |
| IL-9 | 6 (6%) | 62 | 1 | 28 |  | 6 (7%) | 57 |  | 28 |  |
| IL-1b | 53 (55%) | 10 |  | 34 |  | 46 (51%) | 12 |  | 32 |  |
| IL-2 | 51 (53%) | 21 |  | 25 |  | 46 (51%) | 30 |  | 15 |  |
| IL-3 | 15 (15%) | 45 |  | 37 |  | 10 (11%) | 46 |  | 35 |  |
| IL-4 | 42 (43%) | 44 |  | 11 |  | 38 (42%) | 47 |  | 6 |  |
| IL-5 | 22 (23%) | 32 |  | 43 |  | 18 (20%) | 28 |  | 45 |  |
| IL-6 | 72 (74%) | 10 | 7 | 7 | 1 | 51 (56%) | 16 | 7 | 17 |  |
| IL-7 | 43 (44%) | 37 |  | 15 | 2 | 33 (36%) | 44 |  | 14 |  |
| IL-8 | 91 (94%) |  | 5 | 1 |  | 86 (95%) |  | 3 | 2 |  |
| IP-10 | 71 (73%) | 1 | 16 | 9 |  | 73 (80%) |  | 11 | 7 |  |
| MCP-1 | 71 (73%) |  | 11 | 15 |  | 69 (76%) |  | 5 | 17 |  |
| MIP-1a | 79 (81%) | 1 | 5 | 12 |  | 71 (78%) |  | 4 | 16 |  |
| MIP-1b | 97 (100%) |  |  |  |  | 91 (100%) |  |  |  |  |
| RANTES | 97 (100%) |  |  |  |  | 90 (99%) |  |  | 1 |  |
| TNFa | 94 (97%) |  |  | 3 |  | 81 (89%) |  |  | 10 |  |
| VEGF | 54 (56%) | 32 |  | 11 |  | 45 (49%) | 32 |  | 14 |  |
| Eotaxin-2 | 97 (100%) |  |  |  |  | 89 (98%) | 1 |  | 1 |  |
| MCP-2 | 87 (90%) |  | 2 | 8 |  | 84 (92%) |  |  | 7 |  |
| MCP-4 | 46 (47%) | 2 |  | 45 | 4 | 30 (33%) | 7 |  | 53 | 1 |
| I-309 | 93 (96%) | 4 |  |  |  | 87 (96%) | 4 |  |  |  |
| IL-16 | 95 (98%) |  |  |  | 2 | 89 (98%) | 1 |  | 1 |  |
| TARC | 86 (89%) |  |  | 8 | 3 | 82 (90%) |  |  | 8 | 1 |
| Eotaxin-3 | 53 (55%) | 44 |  |  |  | 45 (49%) | 46 |  |  |  |
| LIF | 4 (4%) | 33 |  | 54 | 6 | 7 (8%) | 45 |  | 37 | 2 |
| TPO | 5 (5%) | 49 |  | 42 | 1 | 5 (5%) | 48 |  | 38 |  |
| SCF | 3 (3%) | 68 |  | 26 |  | 3 (3%) | 66 |  | 22 |  |
| TSLP | 2 (2%) | 54 |  | 39 | 2 | 1 (1%) | 53 |  | 37 |  |
| IL-33 | 1 (1%) | 51 |  | 44 | 1 | 1 (1%) | 53 |  | 37 |  |
| IL-20 | 38 (39%) | 28 |  | 29 | 2 | 37 (41%) | 29 |  | 24 | 1 |
| IL-21 | 1 (1%) | 70 |  | 23 | 3 | 1 (1%) | 66 |  | 21 | 3 |
| IL-23 | 20 (21%) | 51 |  | 26 |  | 16 (18%) | 48 |  | 27 |  |
| TRAIL | 3 (3%) | 30 |  | 64 |  | 5 (5%) | 35 |  | 51 |  |
| SDF-1a+b | 35 (36%) | 41 |  | 21 |  | 32 (35%) | 35 |  | 24 |  |
| ENA-78 | 91 (94%) | 1 |  | 5 |  | 85 (93%) | 4 |  | 2 |  |
| MIP1-d | 6 (6%) | 60 |  | 29 | 2 | 8 (9%) | 59 |  | 23 | 1 |
| IL-28A | 3 (3%) | 82 |  | 12 |  | 4 (4%) | 78 |  | 9 |  |

Values are shown as number (%).

OOR< = fluorescence under the detection limit; OOR> = fluorescence exceeds of detection limit; * = sample value extrapolated; *** = sample missing

Cytokine concentrations measured by multiplex ELISA using fluorescence from PBMC culture medium

**Supplementary Table 2**. Minimum limits of quantification of the cytokine plates (pg/ml)

| Cytokine plate (LLOQ) | Median | IQR |
| --- | --- | --- |
| EGF | 3.20 | 3.20-3.21 |
| FGF-2 | 20.7 | 15.6-80.0 |
| Eotaxin | 3.25 | 3.23-3.28 |
| TGF-a | 3.20 | 3.19-3.20 |
| G-CSF | 17.7 | 3.96-47.6 |
| GM-CSF | 3.19 | 3.05-3.23 |
| Fractalkine | 16.5 | 13.3-18.4 |
| IFNa2 | 2.92 | 2.72-3.08 |
| IFNg | 3.22 | 3.19-3.26 |
| IL-10 | 3.20 | 3.19-3.21 |
| MCP-3 | 3.36 | 3.28-3.48 |
| IL-12P40 | 3.13 | 3.08-3.17 |
| MDC | 3.23 | 3.21-3.28 |
| IL-12P70 | 3.24 | 3.21-3.25 |
| IL-13 | 2.90 | 2.76-3.08 |
| IL-15 | 3.10 | 3.03-3.13 |
| sCD40L | 2.84 | 2.67-3.10 |
| IL-17A | 2.82 | 2.63-3.03 |
| IL-1RA | 2.99 | 2.90-3.13 |
| IL-1a | 3.21 | 3.19-3.24 |
| IL-9 | 3.20 | 3.18-3.22 |
| IL-1b | 3.20 | 3.17-3.22 |
| IL-2 | 3.18 | 3.16-3.25 |
| IL-3 | 3.20 | 3.19-3.22 |
| IL-4 | 3.97 | 2.57-17.9 |
| IL-5 | 3.22 | 3.12-3.23 |
| IL-6 | 3.17 | 3.16-3.22 |
| IL-7 | 2.96 | 2.87-3.17 |
| IL-8 | 3.19 | 3.17-3.20 |
| IP-10 | 15.3 | 13.9-19.5 |
| MCP-1 | 3.22 | 3.21-3.24 |
| MIP-1a | 3.12 | 3.09-3.18 |
| MIP-1b | 3.15 | 2.84-17.3 |
| RANTES | 3.02 | 2.88-3.13 |
| TNFa | 3.20 | 3.16-3.24 |
| VEGF | 422 | 47.8-1510 |
| Eotaxin-2 | 9.78 | 9.73-9.84 |
| MCP-2 | 4.88 | 4.86-4.89 |
| MCP-4 | 9.66 | 9.62-9.75 |
| I-309 | 8.55 | 2.23-9.23 |
| IL-16 | 9.44 | 8.58-10.1 |
| TARC | 0.977 | 0.974-0.979 |
| Eotaxin-3 | 227 | 116-253 |
| LIF | 19.5 | 19.2-19.7 |
| TPO | 48.5 | 47.8-49.6 |
| SCF | 9.62 | 9.53-9.87 |
| TSLP | 9.78 | 9.77-9.79 |
| IL-33 | 19.5 | 19.4-19.6 |
| IL-20 | 48.8 | 47.9-49.7 |
| IL-21 | 19.5 | 19.5-19.5 |
| IL-23 | 47.2 | 44.3-49.0 |
| TRAIL | 9.72 | 9.56-9.77 |
| SDF-1a+b | 96.2 | 95.2-98.0 |
| ENA-78 | 19.5 | 19.4-19.6 |
| MIP1-d | 48.8 | 48.8-48.9 |
| IL-28A | 9.32 | 8.56-9.72 |

Values shown as median (pg/ml), IQR (pq/ml), and SEM (pg/ml)

LLOQ, lower limit of quantification; IQR, interquartile range; SEM, standard error of the mean

All Elisa cytokines analyze plates, and their lower limits of detection and plate variation are included.

**Supplementary Table 3**. Maximum limits of quantification of the cytokine plates (pg/ml)

| Cytokine plate (ULOQ) | Median (pg/ml) | IRQ (pq/ml) |
| --- | --- | --- |
| EGF | 2380 | 1020-8360 |
| FGF-2 | 10000 | 9960-10100 |
| Eotaxin | 378 | 371-383 |
| TGF-a | 452 | 422-1530 |
| G-CSF | 10000 | 10000-10000 |
| GM-CSF | 10000 | 9940-10100 |
| Fractalkine | 10000 | 9960-10100 |
| IFNa2 | 10000 | 10000-10000 |
| IFN-g | 10000 | 9980-10000 |
| IL-10 | 10000 | 9990-10000 |
| MCP-3 | 391 | 384-395 |
| IL-12P40 | 10200 | 10100-10200 |
| MDC | 9310 | 8670-9580 |
| IL-12P70 | 10200 | 10100-10300 |
| IL-13 | 10000 | 10000-10000 |
| IL-15 | 10100 | 10100-10200 |
| sCD40L | 10000 | 7840-10000 |
| IL-17A | 10200 | 10100-10300 |
| IL-1RA | 10100 | 10000-10100 |
| IL-1a | 9940 | 9710-10100 |
| IL-9 | 9740 | 8500-10100 |
| IL-1b | 9960 | 9400-10500 |
| IL-2 | 10100 | 9680-10300 |
| IL-3 | 9920 | 9770-10100 |
| IL-4 | 10000 | 10000-10000 |
| IL-5 | 8090 | 2230-9270 |
| IL-6 | 1960 | 1850-5090 |
| IL-7 | 1960 | 1910-1980 |
| IL-8 | 8500 | 1830-9580 |
| IP-10 | 10000 | 9860-10400 |
| MCP-1 | 8330 | 4950-8970 |
| MIP-1a | 439 | 408-2150 |
| MIP-1b | 10400 | 10200-11100 |
| RANTES | 10400 | 5180-11000 |
| TNFa | 9970 | 9540-10600 |
| VEGF | 10900 | 10300-11700 |
| Eotaxin-2 | 1860 | 619-2450 |
| MCP-2 | 1440 | 306-4110 |
| MCP-4 | 1870 | 652-3050 |
| I-309 | 2000 | 1020-2010 |
| IL-16 | 9390 | 2260-11500 |
| TARC | 941 | 825-1060 |
| Eotaxin-3 | 50000 | 37300-50000 |
| LIF | 20000 | 19800-20600 |
| TPO | 50100 | 49700-51400 |
| SCF | 10100 | 9990-10100 |
| TSLP | 9880 | 9430-10600 |
| IL-33 | 15100 | 4260-18800 |
| IL-20 | 48900 | 45200-54600 |
| IL-21 | 19000 | 4160-22100 |
| IL-23 | 50000 | 50000-50300 |
| TRAIL | 9910 | 9690-10100 |
| SDF-1a+b | 97800 | 25700-110000 |
| ENA-78 | 14500 | 4150-17800 |
| MIP1-d | 3600 | 3140-9290 |
| IL-28A | 10000 | 10000-10000 |

Values shown as median (pg/ml), IQR (pq/ml), and SEM (pg/ml)

ULOQ, upper limit of quantification; IQR, interquartile range; SEM, standard error of the mean

All Elisa cytokines analyze plates, and their upper limits of detection and plate variation are included.

*Detailed version of the PBMC extraction protocol*

Blood samples for peripheral blood mononuclear cell (PBMC) processing (>8 ml) were collected to LH Lithium Heparin tube in two different time points (at the study entry and at the convalescent phase [2-week follow-up]). Both samples were analyzed with identical fashion and using the same procedure. After the blood sample was collected, the samples were placed in a rocking shaker and were slowly rocked in a room temperature until the beginning of PBMC processing, which was performed on the same day.

At the beginning of PBMC processing, the sample was centrifuged at 1200 rpm for 10 minutes in +22°C. Thereafter, the plasma was extracted to 15ml falcon tube and frozen. After the plasma extraction, the remaining cell pellet was moved to 50 ml falcon tube after which a phosphate-buffered saline (PBS) solution was added on the top of the cell pellet (final volume of the mixture 20 ml). The sample-PBS-mixture was then carefully divided into two new 15ml falcon tubes each containing 3 ml of room temperature Ficoll-Plaque™ PLUS (GE Healthcare, Amersham, United Kingdom). The mixture was then centrifuged at 2200 rpm for 22 minutes in +22°C. All the visible PBS was then extracted with pipette and PBMCs from both 15 ml falcon tubes was extracted with Pasteur pipette to a new 50 ml falcon tube. 30 ml of PBS was then added on the top of the PBMCs, and the mixture then centrifuged at 1200 rpm for 10 minutes in +22°C. All visible PBS was then extracted, and solution was diluted with 1 ml of PBS. 10 µl of PBMC-PBS-mixture and 90 µl of TryptanBlue (0.5%) was then mixed in Eppendorf tube, and the cells were placed in a Bürker chamber, and the cells were calculated.

After cell calculation, mixture was then diluted that the final sample would contain 2 million cells per ml. Adequately diluted sample was then carefully divided to a 48-well culture plate (one stimulant per well) containing the stimulants. Each well contained 0.5x10^6 PBMCs (in volume of 250 µl) and stimulant (25 µl) diluted to 225 µl of medium (total volume was 500 µl/well). PBMCs were stimulated with anti-CD3/anti-CD28 ([20 µg/ml / 20 µg/ml; final 1 µg/ml / 1 µg/ml; i.e. 0.5 µg/well] BD Biosciences, Franklin Lakes, NJ, USA) in 5% CO2 incubator for 24h and after that the cell and the medium mixture were collected to 2 ml Eppendorf tubes. The samples were centrifuged in 1200 rpm, 1 min at room temperature after which the supernatants were collected and stored in a -80°C refrigerator.

Later, the supernatants were shipped inside dry ice containers to the Swiss Institute of Allergy and Asthma Research (SIAF), Davos, Switzerland. Upon arrival the samples were still frozen and stored in -80°C refrigerator until analysis. Samples were defrosted right before the analyses and analyzed with Millipore HCYTOMAG-60K-36 and HCYP2MAG-62K-20 assay (Merck KGaA, Darmstadt, Germany) using the Bio-Plex 200 System utilizing the Bio-Plex Manager 6.0 Software (Bio-Rad, Cressier, Switzerland) to perform profiling of 56 different cytokines (Supplementary Table 1-3). Internal quality controls for all analytes were satisfactory.

*Stimulants*

More than 700 vials of anti-CD3/anti-CD28 20μg/ml /20μg/ml (final 1 μg/ml / 1 μg/ml), i.e. 0.5 μg/well (BD Biosciences, Franklin Lakes, NJ, USA) were frozen before study (300 to be used at acute phase, 300 at convalescent phase and 100 for reserve). The stimulants were prepared prior to the study at the same time to ensure that concentration of the stimulants remained stable throughout the study period. Each stimulant was be frozen in 25 μl aliquots in –80 °C and defrosted in room temperature just before use in PBMC cultures (0.5 x 10^6^ cells/well, final volume 0.5 ml).

*Medium*

Medium contained 100 ml RPMI-16400, 10 ml iFBS, 1 ml L-glutamine, 20 µl gentamicin, 2 ml HEPES.

*Cytokine analyses*

Due to the limitations of quantitative multiplex Elisa profiling, a few cytokines did not reach the quantitative limit of detection (i.e., fluorescence was under or exceeded the quantification limit of the assay) (Supplementary Table S3). These values were identified as lower limit of quantification (LLOQ) and upper limit of quantification (ULOQ). Each cytokine found in more than 50% of patient samples within the limit of quantification (values between LLOQ and ULOQ) were included for analysis, thus ensuring that conclusions would not be based on minority of samples (Supplementary Table S1-2). Sample was classified as out of range when signal, i.e., fluorescence fell out of the assay range which was both precise and accurate. Due to aforementioned, of 56 cytokines 29 were later classified as eligible for statistical analyses. Samples under the limit of detection were assigned half the value of the LLOQ (Supplementary Table 1) (1,2), and samples exceeding the upper limit of detection (either reported as “ULOQ” or a numerical extrapolated value greater than the assays upper limit of quantification), the values were set to the ULOQ threshold of the assay (Supplementary Table 2) (3).

*Detailed version of the statistics*

Differences in the baseline characteristics between the groups were analyzed by using two-sample t-test for normally distributed and Mann-Whitney U-test for non-normally distributed data. The normality of distribution was assessed by Kolmogorov-Smirnov test. Categorial variables were analyzed using χ^2^ test or Fisher exact test. Due to positively and negatively skewed distribution, continuous cytokine variables were log_10_ or x² transformed before linear model and negative binominal regression analyses.

Differences between study groups in cytokine expression were first analyzed using Mann-Whitney U test for non-normally distributed data and secondly after log_10_- or x²-transformation using multivariable linear model. The adjustments for analyses included baseline characteristics which significantly differed between the groups (age, and duration of previous symptoms [cough, wheezing, fever]). Backward stepwise method was used for the final adjusted model separately for each cytokine. Only statistically significant baseline characteristics variables (*P* < .05) were included in the final model.

Negative binominal regression was used to analyze the effects of viral group (RV vs RV-HBoV1) and cytokine expression on duration of hospitalization. Group × cytokine level interaction effect was included in models and if a statistically significant interaction was found, the cytokine level effect was estimated separately in the RV and the RV-HBoV1 groups. If the interaction was not statistically significant, the effects for the group and the cytokine level were estimated from the main effects model.

A two-sided P value < .05 was considered statistically significant. Data analyses were made using JMP software (version 13.1.0, SAS Institute, Cary, NC, USA).

**Supplementary Table 4.** Differences in cytokine expression levels at study entry, and the convalescent phase

| Cytokine | Timing | RV  n(acute) = 47  n(convalescent) = 20  n(difference) = 17 | RV-HBoV1  n(acute) = 9  n(convalescent) = 4  n(difference) = 4 | p-value, univariate | p-value, multivariate | Adjustments |
| --- | --- | --- | --- | --- | --- | --- |
| EGF | Acute  Convalescent  Difference | 3.50 (1.6–4.3)  -  - | 1.6 (1.6-4.2)  -  - | .20  -  - | .13  -  - | -  -  - |
| G-CSF | Acute  Convalescent  Difference | 10 (7.9–32)  10 (8.9–640)  3.1 (-4.1–570) | 8.8 (8.8–11)  8.9 (4.9-10)  0 (-4.6–0) | .68  .18  .17 | .27  .15  .42 | 1  -  - |
| GM-CSF | Acute  Convalescent  Difference | 12 (1.6–110)  -  - | 1.6 (1.6–16)  -  - | .14  -  - | .15  -  - | -  -  - |
| Fractalkine | Acute  Convalescent  Difference | 14 (8.3–28)  15 (9.3–21)  1.1 (-6.4–8.2) | 13 (7.8–21)  9.9 (3.6–15)  -3.3 (-18–730) | .64  .11  .53 | .59  **.04**  .32 | -  -  - |
| IFNa2 | Acute  Convalescent  Difference | 5.1 (3.0–68)  2.1 (1.5–5.0)  -2.1 (-34–2.2) | 9.0 (1.5–180)  1.5 (1.5–1.5)  -3.8 (-250–0) | .79  .08  .65 | .67  .21  .37 | -  -  - |
| IFNg | Acute  Convalescent  Difference | 9.5 (1.5–81)  12 (1.6–390)  0.15 (-11–260) | 1.6 (1.5–16)  1.2 (1.2–96)  0.01 (-23–94) | .37  .18  .62 | .20  .25  .85 | -  -  - |
| IL-10 | Acute  Convalescent  Difference | 37 (5.3–130)  52 (7.1–380)  1.4 (-190–290) | 14 (4.2–230)  4.6 (2.2–120)  0.5 (-210–120) | .81  .16  >.99 | .34  .18  .74 | 1  -  - |
| MCP-3 | Acute  Convalescent  Difference | 300 (120–390)  390 (36–390)  92 (-98–340) | 99 (44–660)  10 (6.2–64)  -170 (-740–(-34)) | .56  **.03**  **.04** | .59  **.01**  .07 | -  2  - |
| MDC | Acute  Convalescent  Difference | 250 (130–1400)  290 (150–1400)  -26 (-1400–680) | 550 (160–6100)  410 (44–1700)  300 (-100–1100) | .42  .49  .37 | .36  .46  .53 | -  -  - |
| IL-13 | Acute  Convalescent  Difference | 5.5 (1.5–20)  3.2 (1.5–13)  0 (-9.0–9.8) | 4.8 (1.4–12)  3.7 (1.5–100)  0.4 (-8.8–100) | .53  .94  .72 | .33  .96  .19 | -  -  3 |
| IL-1RA | Acute  Convalescent  Difference | 97 (28–420)  170 (50–340)  24 (-280–200) | 370 (67–550)  53 (8.0–380)  -120 (-300–42) | .30  .35  .37 | .74  .22  .56 | 1  -  3 |
| IL-1b | Acute  Convalescent  Difference | 3.5 (1.6–25)  25 (1.6–270)  5.0 (0–220) | 1.6 (1.6–4.7)  1.6 (1.6–15)  0 (0–13) | .13  .09  .49 | **.04**  .12  .64 | 1  -  - |
| IL-2 | Acute  Convalescent  Difference | 3.4 (1.6–21)  3.6 (1.6–21)  0 (-7.7–11) | 6.7 (1.6–21)  8.5 (1.6–44)  0 (-15–43) | .90  .97  .75 | .69  .82  .57 | 4  -  - |
| IL-6 | Acute  Convalescent  Difference | 32 (6.8–380)  66 (4.2–2000)  2.4 (-31–2000) | 7.0 (1.6–27)  1.6 (1.6– 36)  -1.9 (-12–34) | .09  .**049**  .62 | .13  .06  .54 | -  -  - |
| IL-8 | Acute  Convalescent  Difference | 1400 (650–2500)  840 (600–1600)  -10 (-1300–960) | 1000 (430–1800)  240 (120–660)  -510 (-1400–(-47)) | .51  **.02**  .37 | .63  **.02**  .21 | -  -  - |
| IP-10 | Acute  Convalescent  Difference | 1200 (340–10000)  810 (170–3000)  -380 (-8000–430) | 6600 (350–10000)  650 (180–1200)  -3200 (-8100–440) | .18  .54  .86 | .28  .54  .99 | -  -  - |
| MCP-1 | Acute  Convalescent  Difference | 6900 (4200–8300)  6700 (4300–8300)  0 (-3800–2700) | 7400 (3400–8300)  3300 (1100–7200)  -1100 (-3100–3700) | .83  .20  .79 | .91  .24  .54 | -  -  3 |
| MIP-1a | Acute  Convalescent  Difference | 64 (10–440)  180 (3.5–550)  2.2 (-29–430) | 15 (4.1–56)  6.6 (4.0–370)  -1.7 (-23–370) | .14  .70  .86 | .12  .38  .80 | -  -  - |
| MIP-1b | Acute  Convalescent  Difference | 210 (58–940)  430 (48–1300)  150 (-300–640) | 92 (19–310)  30 (17–280)  5.7 (-350–260) | **.03**  .06  .53 | **.03**  .07  .82 | -  -  - |
| RANTES | Acute  Convalescent  Difference | 300 (120–680)  250 (69–800)  -130 (-320–300) | 110 (47–260)  53 (22–530)  -86 (-260–490) | **.03**  .16  .86 | **.001**  .15  .65 | 1,3  2  - |
| TNFa | Acute  Convalescent  Difference | 65 (25–1100)  420 (11–1300)  140 (-50–1100) | 33 (14–69)  12 (4.9–620)  4.2 (-28–600) | .14  .24  .86 | **.04**  .23  .93 | 1  -  - |
| VEGF | Acute  Convalescent  Difference | 63 (12–210)  -  - | 210 (19–210)  -  - | .49  -  - | .15  -  - | 2  -  - |
| Eotaxin-2 | Acute  Convalescent  Difference | 740 (640–980)  770 (450–1100)  -52 (–340–320) | 960 (320-1200)  420 (130–650)  -330 (-660–(-140)) | *.62*  .12  .13 | .86  .97  .39 | -  2-4  - |
| MCP-2 | Acute  Convalescent  Difference | 610 (200–1200)  230 (120–560)  -360 (-770–(-70)) | 400 (180–1000)  290 (79–970)  -440 (-680–790) | .69  >.99  .65 | .63  .70  .50 | -  -  - |
| I-309 | Acute  Convalescent  Difference | 32 (19–67)  24 (14–41)  -8.4 (-20–7.8) | 24 (13–46)  22 (7.2–38)  -3.4 (-17–(-0.2)) | .31  .54  .65 | .11  .41  -85 | 1  -  - |
| IL-16 | Acute  Convalescent  Difference | 66 (50–90)  47 (34–73)  -14 (–49–4.7) | 63 (48–83)  71 (39–220)  2.0 (–160–170) | .70  .25  .64 | .88  .19  .61 | -  -  - |
| TARC | Acute  Convalescent  Difference | 4.4* (3.3–5.9)  4.1 (2.4–7.8)  -0.96 (-2.4–2.5) | 1.9* (1.0–3.6)  2.0 (1.9–5.7)  -0.4 (-2.5–5.2) | .19  .25  >.99 | **.02**  .51  .19 | 1  -  - |
| Eotaxin-3 | Acute  Convalescent  Difference | 110 (110–220)  -  - | 110 (110–130)  -  - | .15  -  - | .54  -  - | -  -  - |
| ENA-78 | Acute  Convalescent  Difference | 900 (170–2200)  190 (65–1800)  -160 (-890–68) | 150 (25–860)  78 (23–150)  -96 (-870–18) | .054  .18  .86 | **.007**  .62  .79 | 2  2,3  - |

Acute sample, samples drawn at the study entry; Convalescent sample, samples drawn at the 2-week follow-up.

Values are shown as medians (interquartile range). Data were analyzed by Mann-Whitney U-test, and by multivariable linear models. Log_10_- (for all other variables) or x^2^-transformed (for MCP-1) cytokine expression levels were used in linear models The adjustments for immunologic analyses included baseline characteristics that significantly differed between the groups (Age = 1, weight = 2, duration of previous symptoms (rhinitis = 3, cough = 4, wheezing =5, fever = 6), and B-Eos = 7 at entry). A backward stepwise method was used for the final adjusted model separately for each cytokine. Only statistically significant baseline characteristics variables (*P* < .05) were included in the final model.

* Age-adjusted geometric means (95% confidence intervals). Bold text; Statistical significance *P <*.05

**Supplementary Table 5.** The biological mechanisms of significant cytokines

| Abbreviation | Cytokine | Biological mechanism | Expression in respiratory virus infection, asthma, and allergy |
| --- | --- | --- | --- |
| CX3CL1 | Fractalkine | Attraction of monocytes, activated T cells, NK cells, and microglia cells (4,5). | Rhinovirus infection has been noted to increase house dust mite induced CX3CL1 shedding, which may be related to the synergism with virus infection and allergen exposure in worsening of asthma (6) Increased expression in the plasma of patients with asthma or symptomatic allergic rhinitis (7). Upregulation in lungs after segmental allergen exposure in patients diagnosed with allergic asthma (7). |
| IL-1β | Interleukin 1 beta | Induction of proinflammatory proteins, differentiation of Th17 cells (8). | Increased activation in rhinovirus infections (9). Upregulation in lungs during HBoV1 infection (10). Promotes inflammation in patients with asthma and persistent allergic rhinitis (11,12). Increased level in nasal aspirates of asthmatic children without respiratory symptoms than in non-asthmatic asymptomatic children (13). |
| RANTES | Regulated upon Activation, Normal T Cell Expressed and Presumably Secreted, CCL5 | Attraction of eosinophils, monocytes, basophils and CD4 T cells. Induction of recruitment of Th2- lymphocytes and eosinophils. Initiation of histamine secretion of basophils. (14-17) | Increased expression in RV-infections compared to RSV and adenovirus infections (18). Upregulation in lungs during HBoV1 infection (10). RANTES is detected in respiratory secretions of asthmatic patients compared to non-asthmatic controls (19). Potential therapeutic effect in alleviation of allergic asthma by increasing the amount of regulatory T cells, by converting inflammatory eosinophils into resident ones and by increasing IL-10 levels in the lung (20). |
| TNF-α | Tumor necrosis factor alpha | Activation of inflammatory cells leading to the release of other proinflammatory cytokines such as IL-8 and RANTES (21). Increase in airway hyperresponsiveness via direct effect on airway smooth muscle (22). | Increased expression in RV infection (23). Detected but lesser expression in HBoV1 bronchiolitis compared to RSV bronchiolitis (24). Increased expression is related to childhood wheezing (25) and the development of asthma (26). |
| TARC | Thymus and activation-regulated chemokine, CCL17 | Attraction of new TARC producing mature dendritic cells and Th2 cells (27). | RV has been detected to increase TARC production measured from serum and nasal fluids (28,29). In RV group, lower expression of TARC during child’s first wheezing episode was related to greater occurrence of a new wheezing episode within two months (30). Upregulation in lungs during HBoV1 infection (10). Significantly higher increase in nasal lining fluid TARC levels in asthmatic patients infected with RV compared to non-asthmatic controls (29). In atopic dermatitis, higher TARC level correlates with the severity of the disease (31,32). |
| ENA-78 | Epithelial-derived neutrophil-activating peptide 78, CXCL5 | Neutrophil attraction and activation via eosinophils, angiogenic properties (33). | RV infection induced higher ENA-78 levels in nasal secretions among asthmatic patients compared to healthy controls (34). In a mouse study, inhibition of ENA-78 in RV-induced asthma exacerbation led to decreased hyperactivity of airways, mucus secretion and collagen disposition (35). |
| MCP-3 | monocyte chemoattractant protein 3, CCL7 | Attraction of monocytes, NK cells and natural killer cells (36,37,38). Recruitment and mobilization of monocytes to inflammatory sites (39,40). | Increased nasal aspirate MCP-3 levels in children suffering acute respiratory infection symptoms compared to asymptomatic children (39). Mice studies suggest that blocking of MCP-3 could have therapeutic effect on respiratory infections and virus induced asthma exacerbations (41). |
| IL-8 | interleukin 8 | Activation of the inflammatory cells by the recruitment of neutrophils, mononuclear phagocytes, mast cells and T cells (42-44). | Increased expression in RV-infections compared to RSV, adenovirus and HBoV1 infections (18,26). IL-8 acts as a contributor to neutrophil activation in virus-induced airway obstruction (45). Increased level in nasal aspirates and serum of asthmatic children without respiratory symptoms than in non-asthmatic asymptomatic children (13,44,46). |
| MIP-1β | macrophage inflammatory protein 1 beta, CCL4 | Attraction of monocytes, T lymphocytes, immature dendritic cells, and natural killer cells (47). | Increased expression in nasopharyngeal aspirates during acute viral respiratory infection in asthmatic children. Discovered positive correlation between MIP-1β levels and infectious lower respiratory tract symptoms. (48)  Higher MIP-1β levels in children suffering from recurrent wheezing than children without history of recurrent wheezing (49). |

**Supplementary Table 6.** Association between cytokine expression and severity of acute illness.

| Outcome  Duration of hospitalization  Log_10_-transformed cytokine level |  |  |  |  |  |
| --- | --- | --- | --- | --- | --- |
|  | Group effect  RV vs RV-HBoV1    Estimate  (95% CI) *P* | | Cytokine effect  Expression of cytokine  Estimate  (95% CI) *P* | | Group × cytokine interaction effect  *P* |
|  |  |  |  |  |  |
| EGF | * | * | 1.090^*^ (0.629, 1.883)  0.192^†^ (0.064, 0.579) | .76^§^  **.003**^#^ | **.043** |
| G-CSF | 0.703^‡^ (0.471, 1.051) | .09 | 1.021^¶^ (0.790, 1.960) | .88 | .08 |
| GM-CSF | 0.740^‡^ (0.482, 1.136) | .17 | 0.972^¶^ (0.818, 1.153) | .74 | .32 |
| Fractalkine | 0.717^‡^ (0.481, 1.069) | .10 | 0.809^¶^ (0.548, 1.194) | .29 | .45 |
| IFNa2 | 0.701^‡^ (0.471, 1.044) | .08 | 0.958^¶^ (0.804, 1.140) | .63 | .13 |
| IFN-g | 0.705^‡^ (0.468, 1.060) | .09 | 1.004^¶^ (0.857, 1.175) | .96 | .06 |
| IL-10 | 0.714^‡^ (0.475, 1.073) | .11 | 0.979^¶^ (0.820, 1.170) | .82 | .11 |
| MCP-3 | 0.704^‡^ (0.474, 1.044) | .08 | 1.165^¶^ (0.852, 1.594) | .34 | .35 |
| MDC | 0.711^‡^ (0.474, 1.065) | .10 | 0.981^¶^ (0.798, 1.207) | .86 | .06 |
| IL-13 | 0.677^‡^ (0.451, 1.016) | .06 | 1.084^¶^ (0.900, 1.306) | .39 | .97 |
| IL-1RA | 0.706^‡^ (0.474, 1.052) | .09 | 0.996^¶^ (0.817, 1.213) | .97 | .51 |
| IL-1b | 0.675^‡^ (0.447, 1.021) | .06 | 1.070^¶^ (0.891, 1.129) | .47 | ****** |
| IL-2 | 0.698^‡^ (0.464, 1.052) | .09 | 1.026^¶^ (0.823, 1.278) | .82 | .06 |
| IL-6 | 0.711^‡^ (0.474, 1.065) | .10 | 0.981^¶^ (0.798, 1.207) | .86 | .08 |
| IL-8 | 0.691^‡^ (0.458, 1.043) | .08 | 1.071^¶^ (0.767, 1.495) | .69 | .96 |
| IP-10 | 0.714^‡^ (0.479, 1.063) | .10 | 1.048^¶^ (0.878, 1.250) | .60 | .36 |
| MCP-1 | 0.655^‡^ (0.425, 1.008) | .054 | 1.427^¶^ (0.642, 3.167) | .38 | .052 |
| MIP-1a | 0.678^‡^ (0.448, 1.027) | .07 | 1.050^¶^ (0.906, 1.217) | .52 | .09 |
| MIP-1b | * | * | 1.200^*^ (0.958, 1.503)  0.588^†^ (0.370, 0.933) | .11^§^  **.024**^#^ | **.027** |
| RANTES | 0.680^‡^ (0.452, 1.023) | .06 | 1.107^¶^ (0.844, 1.451) | .46 | .10 |
| TNFa | 0.651^‡^ (0.432, 0.982) | **.04** | 1.117^¶^ (0.938, 1.331) | .21 | .57 |
| VEGF | 0.757^‡^ (0.511, 1.120) | .16 | 1.212^¶^ (0.979, 1.499) | .08 | .63 |
| Eotaxin-2 | 0.712^‡^ (0.480, 1.058) | .09 | 0.827^¶^ (0.540, 1.270) | .38 | .90 |
| MCP-2 | 0.708^‡^ (0.473, 1.060) | .09 | 0.992^¶^ (0.813, 1.211) | .94 | .53 |
| I-309 | 0.711^‡^ (0.480, 1.053) | .09 | 0.823^¶^ (0.592, 1.157) | .27 | .89 |
| IL-16 | 0.638^‡^ (0.417, 0.976) | **.04** | 0.950^¶^ (0.426, 2.120) | .90 | .85 |
| TARC | 0.788^‡^ (0.527, 1.178) | .25 | 0.755^¶^ (0.554, 1.030) | .08 | .23 |
| Eotaxin-3 | 0.723^‡^ (0.488, 1.072) | .11 | 0.714^¶^ (0.410, 1.246) | .24 | .34 |
| ENA-78 | 0.701^‡^ (0.457, 1.075) | .10 | 1.011^¶^ (0.811, 1.261) | .92 | .62 |

Data were analyzed by negative binominal regression. Log_10_- (for all other variables) or x^2^-transformed (for MCP-1) cytokine expression levels were used in the analyses.

CI = confidence interval

^*^ Relative risk; RV group negative binomial regression

^†^ Relative risk; RV-HBoV1 group negative binomial regression

^¶^ Relative risk

^‡^ Binary logistic regression

^§^ Group effect in RV treatment arm

^#^ Group effect in RV-HBoV1 treatment arm

* Due to the significant interactions, the cytokine effect was not estimated using all data. The effect of cytokine is presented separately in the RV and the RV-HBoV1 groups.

**Interaction effect was not estimable due to all values on RV-HBoV1 group at the detection limit

References

1. Sehmi R., Lim H. F., Mukherjee M., Huang,C., Radford K., Newbold P., et al. Benralizumab attenuates airway eosinophilia in prednisone-dependent asthma. *J Allergy Clin Immunol* (2018) 141:1529-1532.e8. doi: 10.1016/J.JACI.2018.01.008

2. Kolbinger F., Loesche C., Valentin,M. A., Jiang X., Cheng Y., Jarvis P., et al. β-Defensin 2 is a responsive biomarker of IL-17A-driven skin pathology in patients with psoriasis. *J Allergy Clin Immunol* (2017) 139:923-932.e8. doi: 10.1016/J.JACI.2016.06.038

3. Laetsch T.W., Myers G.D., Baruchel A., Dietz A.C., Pulsipher M.A., Bittencour, H., et al. Patient-reported quality of life after tisagenlecleucel infusion in children and young adults with relapsed or refractory B-cell acute lymphoblastic leukaemia: a global, single-arm, phase 2 trial. *Lancet Oncol* (2019) 20:1710–1718. doi: 10.1016/S1470-2045(19)30493-0

4. Tsou C.L., Haskell C.A., Charo I.F. Tumor necrosis factor-alpha-converting enzyme mediates the inducible cleavage of fractalkine. *J Biol Chem* (2001);276(48):44622–44626. doi: 10.1074/jbc.M107327200.

5. Cao J., Gan H., Xiao H., Chen H., Jian D., Jian D., et al. Key protein-coding genes related to microglia in immune regulation and inflammatory response induced by epilepsy. *Math Biosci Eng MBE* (2021);18(6):9563–9578. doi: 10.3934/mbe.2021469.

6. Loxham M., Smart D.E., Bedke N.J., Smithers N.P., Filippi I., Blume C., et al. Allergenic proteases cleave the chemokine CX3CL1 directly from the surface of airway epithelium and augment the effect of rhinovirus. *Mucosal Immunol* (2018);11(2):404–414. doi: 10.1038/mi.2017.63.

7. Rimaniol A.C., Till S.J., Garcia G., Capel F., Godot V., Balabanian K., et al. The CX3C chemokine fractalkine in allergic asthma and rhinitis. *J Allergy Clin Immunol* (2003);112(6):1139–46. doi: 10.1016/j.jaci.2003.09.041.

8. Acosta-Rodriguez E.V., Napolitani G., Lanzavecchia A., Sallusto F. Interleukins 1beta and 6 but not transforming growth factor-beta are essential for the differentiation of interleukin 17-producing human T helper cells. *Nat Immunol.* 2007;8(9):942–949. doi: 10.1038/ni1496.

9. Han M., Bentley J.K., Rajput C., Lei J., Ishikawa T., Jarman C.R., et al. Inflammasome activation is required for human rhinovirus-induced airway inflammation in naive and allergen-sensitized mice. *Mucosal Immunol* (2019);12(4):958–968. doi: 10.1038/s41385-019-0172-2.

10. Khalfaoui S., Eichhorn V., Karagiannidis C., Bayh I., Brockmann M., Pieper M., et al. Lung infection by human bocavirus induces the release of profibrotic mediator cytokines in vivo and in vitro. *PLoS One* (2016);11(1):e0147010. doi: 10.1371/journal.pone.0147010.

11. Midulla F., Villani A., Panuska J.R., Dab I., Kolls J.K., Merolla R., et al. Respiratory syncytial virus lung infection in infants: immunoregulatory role of infected alveolar macrophages. *J Infect Dis* (1993);168(6):1515–9. doi: 10.1093/infdis/168.6.1515.

12. Han M.W., Kim S.H., Oh I., Kim Y.H., Lee J. Serum IL-1β can be a biomarker in children with severe persistent allergic rhinitis. *Allergy Asthma Clin Immunol* (2019);18;15:58. doi: 10.1186/s13223-019-0368-8.

13. Jazaeri S., Goldsmith A.M., Jarman C.R., Lee J., Hershenson M.B., Lewis T.C. Nasal interferon responses to community rhinovirus infections are similar in controls and children with asthma. *Ann Allergy Asthma Immunol* (2021);126(6):690–695. doi: 10.1016/j.anai.2021.01.023.

14. Alam R., Stafford S., Forsythe P., Harrison R., Faubion D., Lett-Brown M.A., et al. RANTES is a chemotactic and activating factor for human eosinophils. *J Immunol* (1993). Apr 15; 150(8 Pt 1):3442–8

15. Schall T.J., Bacon K., Toy K.J., Goeddel D.V. Selective attraction of monocytes and T lymphocytes of the memory phenotype by cytokine RANTES. *Nature* (1990) 347:669–71. doi: 10.1038/347669a0.

16. Bacon K.B., Premack B.A., Gardner P., Schall T.J. Activation of dual T cell signaling pathways by the chemokine RANTES. *Science* (1995); 269:1727–1730. doi: 10.1126/science.7569902.

17. Kuna P., Reddigari S.R., Schall T.J., Rucinski D., Viksman M.Y., Kaplan A.P. RANTES, a monocyte and T lymphocyte chemotactic cytokine releases histamine from human basophils. *J Immunol* (1992);149:636–642.

18. Chun Y.H., Park J.Y., Lee H., Kim H.S., Won S., Joe H.J., et al. Rhinovirus-infected epithelial cells produce more IL-8 and RANTES compared with other respiratory viruses. *Allergy Asthma Immunol Res* (2013);5:216–223. doi: 10.4168/aair.2013.5.4.216.

19. Conti P., DiGioacchino M. MCP-1 and RANTES are mediators of acute and chronic inflammation*. Allergy Asthma Proc.* (2001); 22(3):133–7. doi: 10.2500/108854101778148737.

20. Li N., Mirzakhani H., Kiefer A., Koelle J., Vuorinen T., Rauh M., et al. Regulated on Activation, Normal T cell Expressed and Secreted (RANTES) drives the resolution of allergic asthma. *iScience* (2021);24(10):103163. doi: 10.1016/j.isci.2021.103163. doi: 10.1016/j.isci.2021.103163.

21. Babu K.S., Davies D.E., Holgate S.T. Role of tumor necrosis factor alpha in asthma. *Immunol Allergy Clin North Am* (2004); 24(4):583–97. doi: 10.1016/j.iac.2004.06.010.

22. Amrani Y., Chen H., Panettieri R.A. Jr. Activation of tumor necrosis factor receptor 1 in airway smooth muscle: a potential pathway that modulates bronchial hyper-responsiveness in asthma? *Respir Res* (2000); 1(1): 49–53. doi: 10.1186/rr12.

23. Gern J.E., Dick E.C., Lee W.M., Murray S., Meyer K., Handzel Z.T., et al. Rhinovirus enters but does not replicate inside monocytes and airway macrophages. *J. Immunol* (1996);156(2):621–627.

24. Chung J.Y., Han T.H., Kim J.S., Kim S.W., Park C.G., Hwang E.S. Th1 and Th2 cytokine levels in nasopharyngeal aspirates from children with human bocavirus bronchiolitis. *J. Clin. Virol.* (2008); 43:223–225. doi: 10.1016/j.jcv.2008.06.008.

25. Balfour-Lynn I.M., Valman H.B., Wellings R., Webster A.D., Taylor G.W., Silverman M. Tumor necrosis factor–alpha and leukotriene E4 production in wheezy infants. *Clin Exp Allergy* (1994); 24(2):121–126. doi: 10.1111/j.1365-2222.1994.tb00207.x.

26. Lukkarinen H., Söderlund-Venermo M., Vuorinen T., Allander T., Hedman K., Simell O., et al. Human bocavirus 1 may suppress rhinovirus-associated immune response in wheezing children. *J Allergy Clin Immun* (2014); 133: 256–258. doi: 10.1016/j.jaci.2013.10.014.

27. Sallusto F., Lenig D., Mackay C.R., Lanzavecchia A. Flexible programs of chemokine receptor expression on human polarized T helper 1 and 2 lymphocytes. *J Exp Med* (1998);187(6):875–883. doi: 10.1084/jem.187.6.875.

28. Hansel T.T., Tunstall T., Trujillo-Torralbo M.B., Shamji B., Del-Rosario A., Dhariwal J., et al. A comprehensive evaluation of nasal and bronchial cytokines and chemokines following experimental rhinovirus infection in allergic asthma: Increased interferons (IFN-γ and IFN-λ) and type 2 inflammation (IL-5 and IL-13). *EBioMedicine* (2017);19:128–138. doi: 10.1016/j.ebiom.2017.03.033.

29. Nikonova A., Khaitov M., Jackson D.J., Traub S., Trujillo-Torralbo M.B., Kudlay D.A., et al. M1-like macrophages are potent producers of anti-viral interferons and M1-associated marker-positive lung macrophages are decreased during rhinovirus-induced asthma exacerbations. *EBioMedicine* (2020);54:102734. doi: 10.1016/j.ebiom.2020.102734.

30. Hurme P., Komulainen M., Tulkki M., Leino A., Rückert B., Turunen R., et al. Cytokine expression in rhinovirus- vs. respiratory syncytial virus-induced first wheezing episode and its relation to clinical course. *Front Immunol* (2022);13:1044621. doi: 10.3389/fimmu.2022.1044621

31. Kakinuma T., Nakamura K., Wakugawa M., Mitsui H., Tada Y., Saeki H., et al. Thymus and activation-regulated chemokine in atopic dermatitis: Serum thymus and activation-regulated chemokine level is closely related with disease activity. *J Allergy Clin Immunol* (2001);107(3):535–41. doi: 10.1067/mai.2001.113237.

32. Nakazato J., Kishida M., Kuroiwa R., Fujiwara J., Shimoda M., Shinomiya N. Serum levels of Th2 chemokines, CCL17, CCL22, and CCL27, were the important markers of severity in infantile atopic dermatitis. *Pediatr Allergy Immunol* (2008);19(7):605–13. doi: 10.1111/j.1399-3038.2007.00692.x.

33. Walz A., Schmutz P., Mueller C., Schnyder-Candrian S. Regulation and function of the CXC chemokine ENA-78 in monocytes and its role in disease. *J Leukoc Biol* (1997);62(5):604–611. doi: 10.1002/jlb.62.5.604.

34. Donninger H., Glashoff R., Haitchi H.M., Syce J.A., Ghildyal R., van Rensburg E., et al. Rhinovirus induction of the CXC chemokine epithelial-neutrophil activating peptide-78 in bronchial epithelium. *J Infect Dis.* (2003);187(11):1809–17. doi: 10.1086/375246.

35. Sokulsky L.A., Garcia-Netto K., Nguyen T.H., Girkin J.L.N., Collison A., Mattes J., et al. A critical role for the CXCL3/CXCL5/CXCR2 neutrophilic chemotactic axis in the regulation of type 2 responses in a model of rhinoviral-induced asthma exacerbation. *J Immunol.* (2020);205(9):2468–2478. doi: 10.4049/jimmunol.1901350.

36. Van Damme J., Proost P., Lenaerts J.P., Opdenakker G. Structural and functional identification of two human, tumor-derived monocyte chemotactic proteins (MCP-2 and MCP-3) belonging to the chemokine family. *J. Exp. Med* (1992);176: 59–65. doi: 10.1084/jem.176.1.59.

37. Allavena P., Bianchi G., Zhou D., van Damme J., Jílek P., Sozzani S., et al. Induction of natural killer cell migration by monocyte chemotactic protein-1, -2 and -3. *Eur J Immunol* (1994); 24: 3233–36. doi: 10.1002/eji.1830241249.

38. Taub D.D., Proost P., Murphy W.J., Anver M., Longo D.L., van Damme J., et al. Monocyte chemotactic protein-1 (MCP-1), -2, and -3 are chemotactic for human T lymphocytes. *J Clin Invest* (1995); 95: 1370–76. doi: 10.1172/JCI117788.

39. Santiago J., Hernández-Cruz J.L., Manjarrez-Zavala M.E., Montes-Vizuet R., Rosete-Olvera D.P., Tapia-Díaz A.M., et al. Role of monocyte chemotactic protein-3 and -4 in children with virus exacerbation of asthma. *European Respiratory Journal* (2008);32:1243–1942. doi: 10.1183/09031936.00085107.

40. Tsou C.L., Peters W., Si Y., Slaymaker S., Aslanian A.M., Weisberg S.P., et al. Critical roles for CCR2 and MCP-3 in monocyte mobilization from bone marrow and recruitment to inflammatory sites. *J. Clin. Invest* (2007); 117: 902–909. doi: 10.1172/JCI29919.

41. Dawson T.C., Beck M.A., Kuziel W.A., Henderson F., Maeda N. Contrasting effects of CCR5 and CCR2 deficiency in the pulmonary inflammatory response to influenza A virus. *Am J Pathol* (2000); 156: 1951–1959. doi: 10.1016/S0002-9440(10)65068-7.

42. Beigelman A., Isaacson-Schmid M., Sajol G., Baty J., Rodriguez O.M., Leege E., et al. Randomized trial to evaluate azithromycin’s effects on serum and upper airway IL-8 levels and recurrent wheezing in infants with respiratory syncytial virus bronchiolitis. *J Allergy Clin Immunol* (2015);135(5):1171-1178.e1. doi: 10.1016/j.jaci.2014.10.001.

43. Wang W., Qu X., Dang X., Shang D., Yang L., Li Y., et al. Human β-defensin-3 induces IL-8 release and apoptosis in airway smooth muscle cells. *Clin Exp Allergy* (2017);47(9):1138–1149. doi: 10.1111/cea.12943.

44. Charrad R., Kaabachi W., Rafrafi A., Berraies A., Hamzaoui K., Hamzaoui A. IL-8 gene variants and expression in childhood asthma. *Lung* (2017);195(6):749–757. doi: 10.1007/s00408-017-0058-6.

45. Sehmi R., Cromwell O., Wardlaw A.J., Moqbel R., Kay A.B.. Interleukin-8 is a chemo-attractant for eosinophils purified from subjects with a blood eosinophilia but not from normal healthy subjects. *Clin Exp Allergy* (1993); 23:1027–1036. doi: 10.1111/j.1365-2222.1993.tb00295.x.

46. Vizmanos-Lamotte G., Moreno-Galdó A., Muñoz X., Gómez-Ollés S., Gartner S., Cruz M.J. Induced sputum cell count and cytokine profile in atopic and non-atopic children with asthma. *Pediatr Pulmonol* (2013);48(11):1062–9. doi: 10.1002/ppul.22769.

47. Menten P., Wuyts A., Van Damme J. Macrophage inflammatory protein-1. *Cytokine Growth Factor Rev.* (2002); 13(6):455–81. doi: 10.1016/s1359-6101(02)00045-x.

48. Lewis T.C., Henderson T.A., Carpenter A.R., Ramirez I.A., McHenry C.L., Goldsmith A.M., et al. Nasal cytokine responses to natural colds in asthmatic children. *Clin Exp Allergy* (2012); 42(12):1734–44. doi: 10.1111/cea.12005.

49. Sugai K., Kimura H., Miyaji Y., Tsukagoshi H., Yoshizumi M., Sasaki-Sakamoto T., et al. MIP-1α level in nasopharyngeal aspirates at the first wheezing episode predicts recurrent wheezing. *J Allergy Clin Immunol* (2016); 137(3):774–81. doi: 10.1016/j.jaci.2015.08.032..
